# Vascular Endothelial Growth Factor Receptor-2 Promotes the Development of the Lymphatic Vasculature

**DOI:** 10.1371/journal.pone.0074686

**Published:** 2013-09-02

**Authors:** Michael T. Dellinger, Stryder M. Meadows, Katherine Wynne, Ondine Cleaver, Rolf A. Brekken

**Affiliations:** 1 Division of Surgical Oncology, Department of Surgery, Hamon Center for Therapeutic Oncology Research, University of Texas Southwestern Medical Center, Dallas, Texas, United States of America; 2 Department of Molecular Biology, University of Texas Southwestern Medical Center, Dallas, Texas, United States of America; 3 Department of Pharmacology, University of Texas Southwestern Medical Center, Dallas, Texas, United States of America; Feinberg Cardiovascular Research Institute, Northwestern University, United States of America

## Abstract

Vascular endothelial growth factor receptor 2 (VEGFR2) is highly expressed by lymphatic endothelial cells and has been shown to stimulate lymphangiogenesis in adult mice. However, the role VEGFR2 serves in the development of the lymphatic vascular system has not been defined. Here we use the *Cre-lox* system to show that the proper development of the lymphatic vasculature requires VEGFR2 expression by lymphatic endothelium. We show that *Lyve-1^wt/Cre^;Vegfr2^flox/flox^* mice possess significantly fewer dermal lymphatic vessels than *Vegfr2^flox/flox^* mice. Although *Lyve-1^wt/Cre^;Vegfr2^flox/flox^* mice exhibit lymphatic hypoplasia, the lymphatic network is functional and contains all of the key features of a normal lymphatic network (initial lymphatic vessels and valved collecting vessels surrounded by smooth muscle cells (SMCs)). We also show that *Lyve-1^Cre^* mice display robust *Cre* activity in macrophages and in blood vessels in the yolk sac, liver and lung. This activity dramatically impairs the development of blood vessels in these tissues in *Lyve-1^wt/Cre^;Vegfr2^flox/flox^* embryos, most of which die after embryonic day14.5. Lastly, we show that inactivation of *Vegfr2* in the myeloid lineage does not affect the development of the lymphatic vasculature. Therefore, the abnormal lymphatic phenotype of *Lyve-1^wt/Cre^;Vegfr2^flox/flox^* mice is due to the deletion of *Vegfr2* in the lymphatic vasculature not macrophages. Together, this work demonstrates that VEGFR2 directly promotes the expansion of the lymphatic network and further defines the molecular mechanisms controlling the development of the lymphatic vascular system.

## Introduction

The lymphatic vasculature transports immune cells, absorbs dietary fats, and regulates tissue fluid homeostasis by returning fluid and macromolecules to the blood vascular system [1]. Insufficiency of the lymphatic vascular system leads to the formation of lymphedema, a condition characterized by massive swelling of affected limbs, fibrosis, and impaired immunity [1]. In humans, mutations in *VEGF-C*, *VEGFR3*, *GJC2*, *GJA1*, *KIF11*, *FOXC2*, *CCBE1*, *SOX18*, *PTPN14*, and *GATA2* have been found in families with inherited forms of lymphedema and cause striking defects in the development of the lymphatic vasculature [2]–[12]. These important clinical observations have fueled efforts to further identify the molecular mechanisms governing the formation of the lymphatic vasculature.

The current model for the development of the mammalian lymphatic vascular system conforms to the centrifugal theory originally proposed by Florence Sabin over 100 years ago [13]. According to this model, lymphatic endothelial cells (LECs) differentiate from blood endothelial cells (BECs) and migrate from veins to form lymph sacs during embryogenesis. Sprouting from these sacs gives rise to an immature lymphatic network which remodels into a hierarchal pattern of capillaries and valved collecting vessels [14]. The centrifugal theory for mammals has been supported by the expression pattern of molecular markers of LECs [15], lineage tracing experiments [16], and by the unique mutant phenotypes of genetically modified mice (reviewed in [17]). Despite these recent advances, the molecular mechanisms driving the expansion of the lymphatic network remain largely unknown.

There is growing evidence that VEGFR2, a receptor tyrosine kinase activated by VEGF-A, -C and -E, stimulates lymphangiogenesis. Overexpression of VEGFR2 ligands in the skin of adult mice and in tumors induces the growth of lymphatic vessels [18]–[21]. Furthermore, VEGF-A has been shown to promote lymphangiogenesis in the corneal micropocket assay in a VEGF-C/-D/-R3 independent manner [21]. To gain a better understanding of how VEGFR2 stimulates lymphangiogenesis, numerous *in vitro* experiments have been performed with LECs. These reports have shown that VEGF-A/VEGFR2 signaling promotes LEC proliferation, migration, and tube formation as well as the permeability of LEC monolayers [22]–[27]. We recently reported that VEGF-A stimulation of LECs leads to the phosphorylation of VEGFR2 on several tyrosine residues (Tyr 951, Tyr 1054, Tyr 1059, Tyr 1175 and Tyr 1214), and promotes protein kinase C dependent phosphorylation of ERK1/2 and PI3-K dependent phosphorylation of Akt [28]. The activation of both of these pathways was required for VEGF-A/VEGFR2-induced proliferation and migration of LECs [28]. These studies have begun to shed light on the molecular pathways and cellular processes activated by VEGFR2 in LECs. However, the role VEGFR2 serves in the development of the lymphatic vasculature has not been explored, in part, because *Vegfr2* knockout mice die before the lymphatic vascular system forms [29]. In the present study, we use the *Cre*-*lox* system to overcome this obstacle and conditionally inactivate *Vegfr2* in LECs to characterize its function in the development of the lymphatic vascular system.

## Results

### Adult Lyve-1^wt/Cre^;Vegfr2^flox/flox^ mice display lymphatic hypoplasia

**Figure 1:**
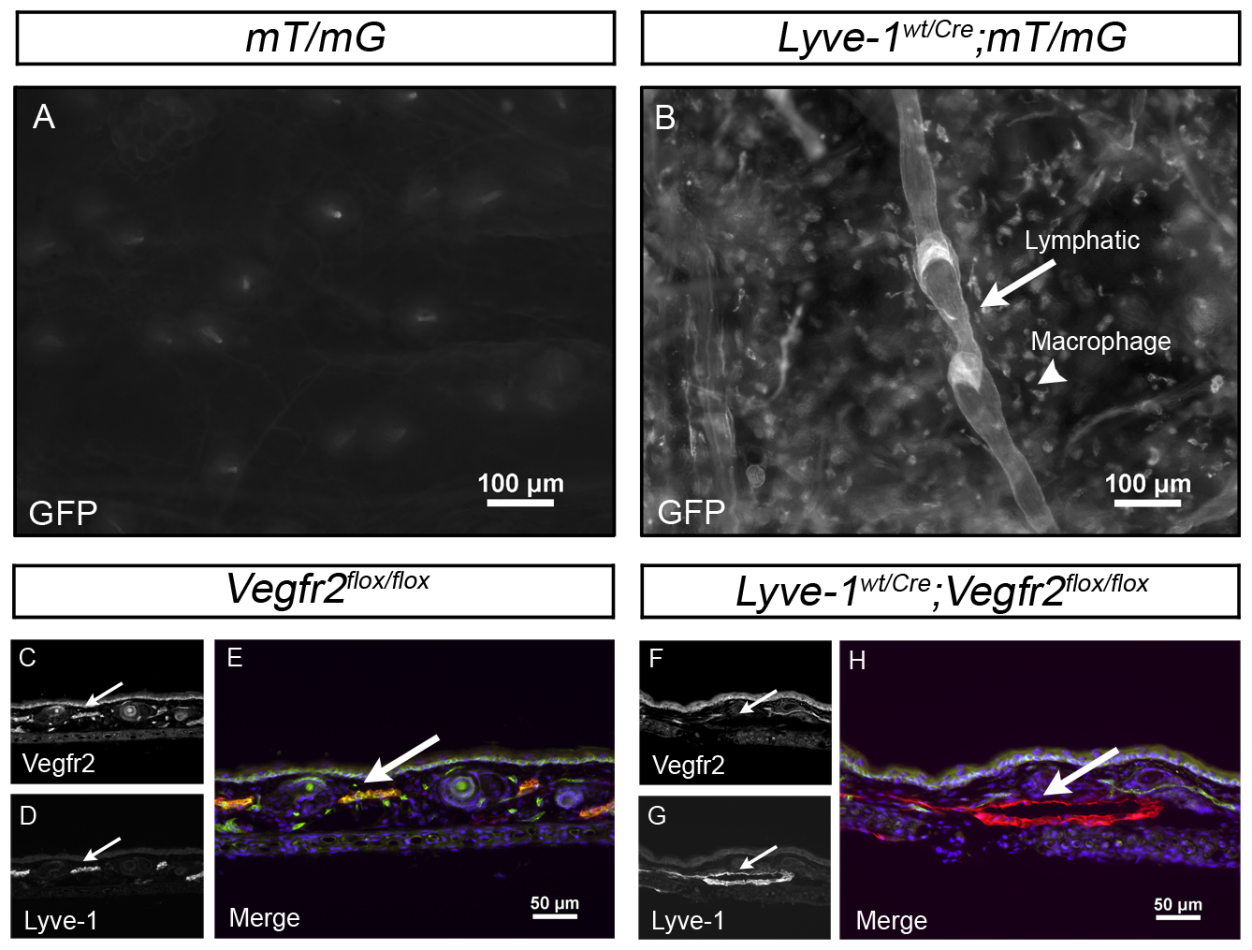
Cre recombinase-mediated deletion of *floxed* sequences in dermal lymphatic vessels. (A,B) Whole-mount preparations of ear skin from *mT/mG* and *Lyve-1^wt/Cre^;mT/mG* mice. GFP is not expressed in the skin of *mT/mG* mice (A) but is expressed by lymphatic vessels and macrophages in the skin of *Lyve-1^wt/Cre^;mT/mG* mice (B). (C-H) Representative images of transverse sections of ear skin from adult *Vegfr2^flox/flox^* mice and *Lyve-1^wt/Cre^;Vegfr2^flox/flox^* mice stained with antibodies against Vegfr2 and Lyve-1. Vegfr2 (C) is expressed by Lyve-1 positive (D) lymphatic vessels in *Vegfr2^flox/flox^* mice (E, arrow). In contrast, Vegfr2 (F) expression is lost by Lyve-1 positive (G) lymphatic vessels in *Lyve-1^wt/Cre^;Vegfr2^flox/flox^* mice (H, arrow).

Lyve-1 is a hyaluronan receptor highly expressed by lymphatic capillaries but not by collecting lymphatic vessels or valves [30], [31]. Although collecting lymphatic vessels and valves do not express Lyve-1, they are thought to arise from Lyve-1 positive LECs [30], [32]. *Lyve-1^Cre^* mice were recently developed to conditionally delete *floxed* (flanking *loxP*) DNA sequences in LECs [33]. However, the removal of *floxed* DNA sequences in collecting lymphatic vessels and valves was not previously analyzed. To further characterize the pattern of *Cre*-mediated recombination in *Lyve-1^Cre^* mice, we crossed this strain with the *mT/mG* reporter strain. *mT/mG* mice possess a GFP reporter whose expression is induced by Cre-mediated recombination [34]. Lymphatic vessels in ear skin whole-mounts from *Lyve-1^wt/Cre^* or *mT/mG* mice did not express GFP. However, *Lyve-1^wt/Cre^;mT/mG* mice displayed strong GFP expression in macrophages as well as in lymphatic capillaries, collecting lymphatic vessels and valves (Figure 1A, 1B). This result confirms previous reports documenting that Lyve-1-negative collecting vessels arise from Lyve-1-positive vessels [30], [32] and indicates that the *Lyve-1^Cre^* mouse can be used to excise *floxed* DNA sequences in LECs that give rise to lymphatic capillaries, collecting lymphatic vessels and valves.

**Figure 2:**
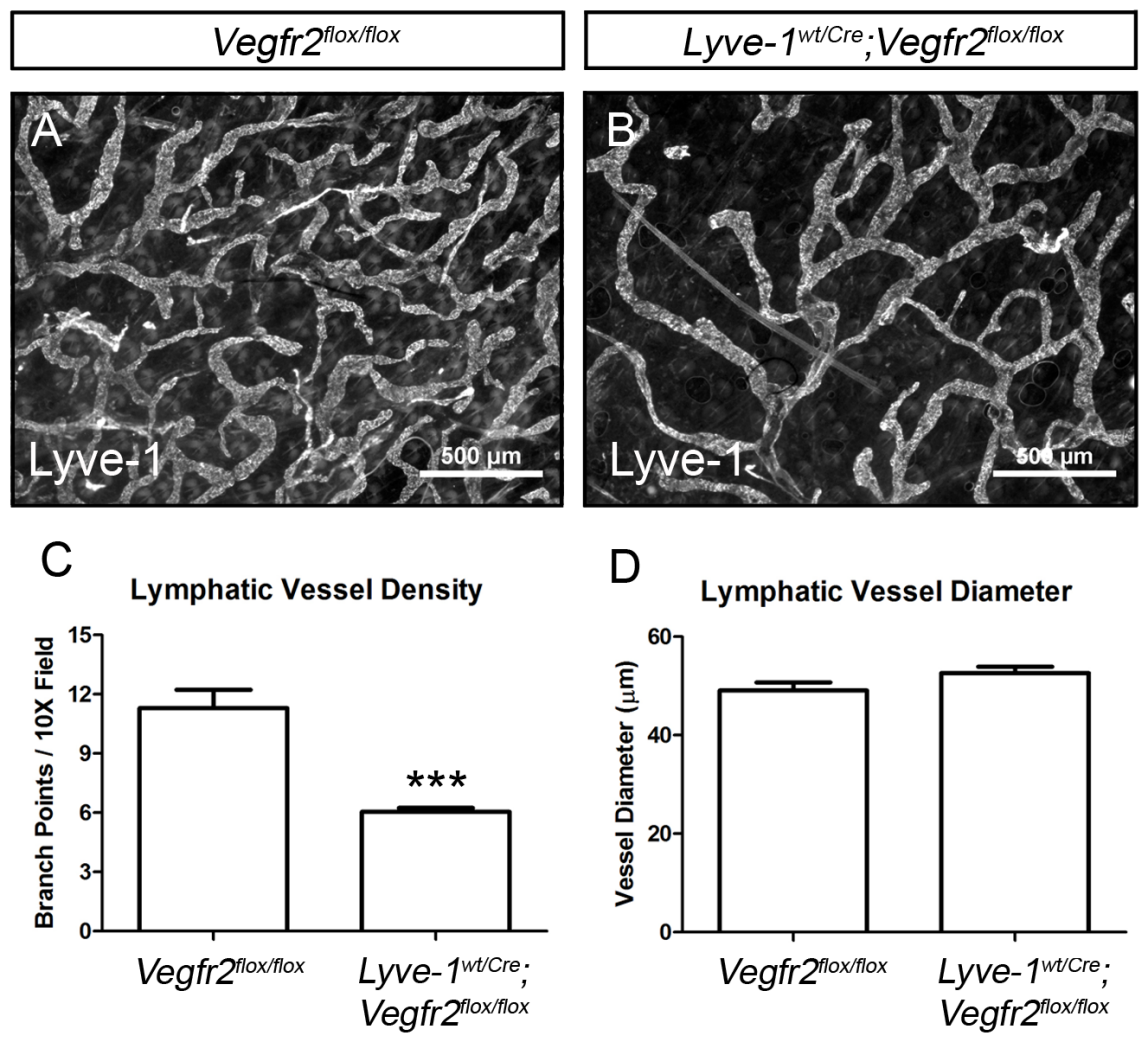
*Lyve-1^wt/Cre^;Vegfr2^flox/flox^* mice exhibit a deficiency of dermal lymphatic vessels. (A,B) Whole-mount immunofluorescence staining of ear skin from *Vegfr2^flox/flox^* and *Lyve-1^wt/Cre^;Vegfr2^flox/flox^* mice for Lyve-1. (C) Quantitative analysis showing that there are significantly fewer lymphatic branch points in the ear skin of *Lyve-1^wt/Cre^;Vegfr2^flox/flox^* mice (6.042±0.198, n = 6) than *Vegr2^flox/flox^* mice (11.29±0.940, n = 6). (D) Lymphatic vessel diameter is not significantly different between *Lyve-1^wt/Cre^;Vegfr2^flox/flox^* (52.65 µm±1.303, n = 6) and *Vegfr2^flox/flox^* mice (49.05 µm±1.675, n = 6). *** indicates P || 0.001.

*Lyve-1^Cre^* mice were bred with Vegfr2 *floxed* mice (*Vegfr2^flox^*) to investigate the role Vegfr2 serves in the development of the lymphatic vasculature. Immunofluorescence staining of sections of adult ear skin with antibodies against Lyve-1 and Vegfr2 revealed that, on average, 98.6% of the Lyve-1-positive LECs lacked Vegfr2 in *Lyve-1^wt/Cre^;Vegfr2^flox/flox^* mice (n = 3 mice; Figure 1C-H). Vegfr2 expression by dermal blood vessels was unaffected in *Lyve-1^wt/Cre^;Vegfr2^flox/flox^* mice. Whole-mount immunofluorescence staining of ear skin for Lyve-1 showed a highly branched network of lymphatic capillaries in *Vegfr2^flox/flox^* mice (Figure 2A). In contrast, *Lyve-1^wt/Cre^;Vegfr2^flox/flox^* mice exhibited a hypoplastic network of lymphatic capillaries (Figure 2B). Quantitative analysis showed that there were significantly fewer lymphatic branch points in *Lyve-1^wt/Cre^;Vegfr2^flox/flox^* mice (6.042±0.198, n = 6) than in *Vegr2^flox/flox^* mice (11.29±0.940, n = 6; Figure 2C). However, lymphatic vessel diameter was not significantly different between *Lyve-1^wt/Cre^;Vegfr2^flox/flox^* (52.65 µm±1.303, n = 6) and *Vegfr2^flox/flox^* mice (49.05 µm±1.675, n = 6; Figure 2D).

### Vegfr2 is not required for the maturation of collecting lymphatic vessels

**Figure 3:**
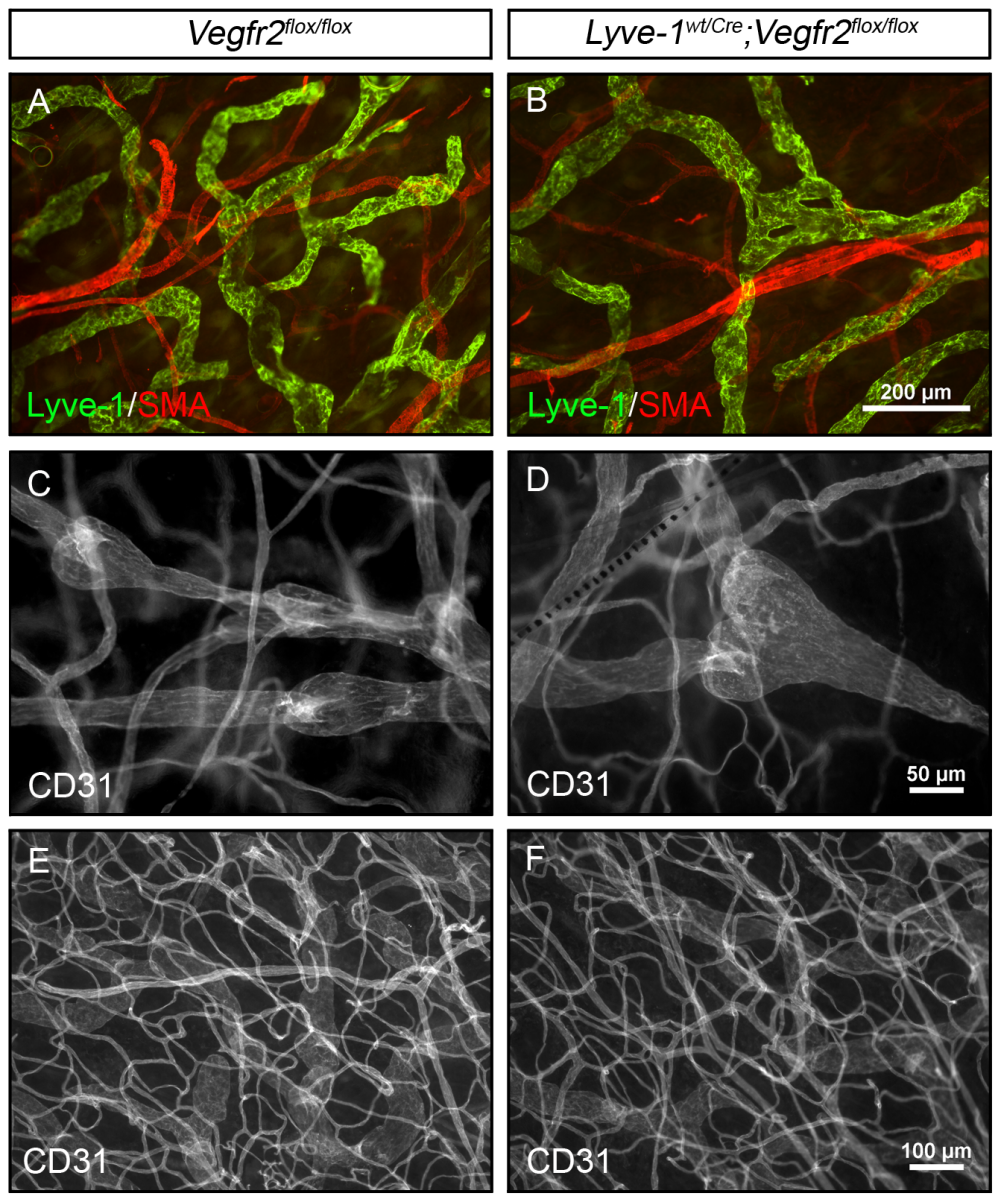
Lymphatic maturation and blood vessel patterning are normal in *Lyve-1^wt/Cre^;Vegfr2^flox/flox^* mice. Whole-mount immunofluorescence staining of ear skin from adult *Vegfr2^flox/flox^* and *Lyve-1^wt/Cre^;Vegfr2^flox/flox^* mice for Lyve-1 (A,B), smooth muscle actin (A,B), and CD31 (C-F). (A,B) Lyve-1 positive lymphatic vessels are not covered by SMA positive cells in *Vegfr2^flox/flox^* or *Lyve-1^wt/Cre^;Vegfr2^flox/flox^* mice. (C,D) CD31 expressing lymphatic valves are present in the collecting vessels of *Vegfr2^flox/flox^* and *Lyve-1^wt/Cre^;Vegfr2^flox/flox^* mice. (E,F) The patterning of CD31 labeled blood vessels is similar for *Vegfr2^flox/flox^* and *Lyve-1^wt/Cre^;Vegfr2^flox/flox^* mice.

Lymphatic capillaries and collecting lymphatic vessels are differentially covered by SMCs. Lyve-1-positive lymphatic capillaries are free of SMCs whereas Lyve-1-negative collecting vessels are covered by SMCs. Interestingly, defects in the patterning of the lymphatic vasculature have been associated with the mislocalization of SMCs on Lyve-1-positive lymphatic vessels [30], [31], [35]. To determine whether the hypoplastic lymphatic network in *Lyve-1^wt/Cre^;Vegfr2^flox/flox^* mice was aberrantly covered by SMCs, we stained ear skin from adult mice for Lyve-1 and smooth muscle actin. This revealed that the Lyve-1-positive lymphatic networks in *Vegfr2^flox/flox^* and *Lyve-1^wt/Cre^;Vegfr2^flox/flox^* mice were not surrounded by SMCs (Figure 3A, 3B; n = 3 for each genotype). However, SMCs were properly associated with Lyve-1-negative vessels in both strains of mice (data not shown).

Next we evaluated whether Vegfr2 is required for formation of lymphatic valves. Whole-mount immunofluorescence staining of adult ear skin for Lyve-1 and CD31 showed that Lyve-1-positive CD31-positive lymphatic capillaries transitioned into Lyve-1-negative CD31-positive collecting lymphatic vessels in *Vegfr2^flox/flox^* and *Lyve-1^wt/Cre^;Vegfr2^flox/flox^* mice. Importantly, CD31-positive collecting lymphatic vessels in *Vegfr2^flox/flox^* and *Lyve-1^wt/Cre^;Vegfr2^flox/flox^* mice possessed normal appearing lymphatic valves consisting of a bulbous region containing intraluminal leaflets (Figure 3C, 3D; n = 4 for each genotype).

The CD31 staining also revealed that the patterning of the blood vasculature was normal in the ear skin of adult *Lyve-1^wt/Cre^;Vegfr2^flox/flox^* mice (Figure 3E, 3F). The number of blood vessel branch points was not significantly different between *Vegfr2^flox/flox^* (94.38±1.679; n = 4) and *Lyve-1^wt/Cre^;Vegfr2^flox/flox^* mice (97.41±0.8079; n = 4). Therefore, *Lyve-1^wt/Cre^;Vegfr2^flox/flox^* mice display a dermal lymphatic vascular phenotype in the absence of a dermal blood vascular phenotype. Together, these results suggest that the lymphatic defect in the skin of *Lyve-1^wt/Cre^;Vegfr2^flox/flox^* mice is not secondary to a blood vessel defect in the skin.

### Lymphatic function is normal in adult Lyve-1^wt/Cre^;Vegfr2^flox/flox^ mice

**Figure 4:**
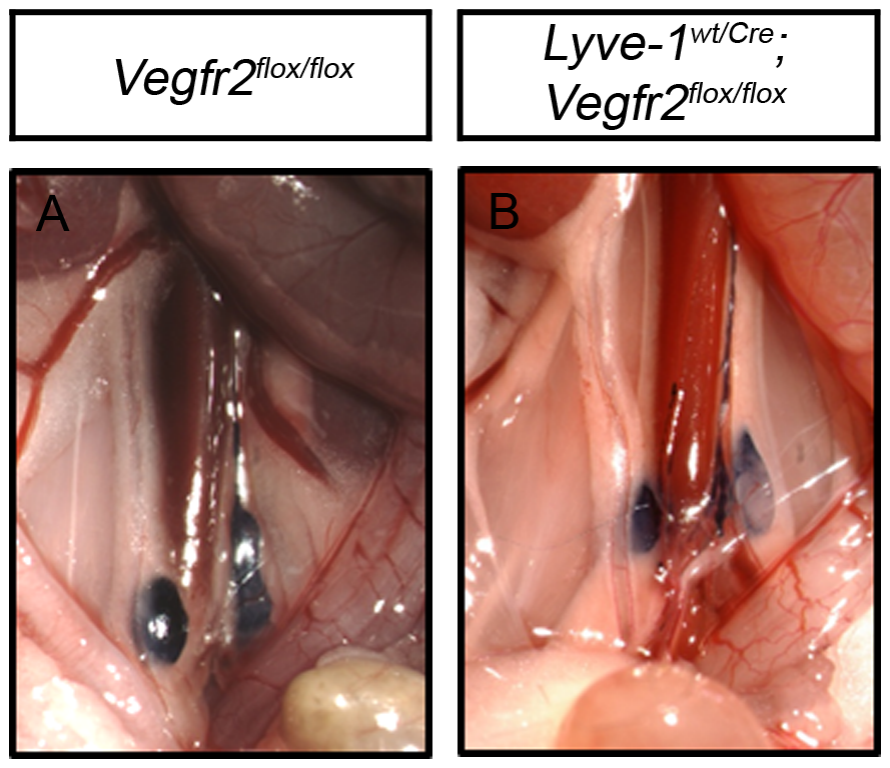
Lymphatic drainage is normal in *Lyve-1^wt/Cre^;Vegfr2^flox/flox^* mice. (A,B) Intradermally administered EBD is transported from the hind paws to the iliac lymph nodes in all *Vegfr2^flox/flox^* (n = 6) and *Lyve-1^wt/Cre^;Vegfr2^flox/flox^* mice (n = 6).

The dramatic reduction in lymphatic vessel density led us to assess lymphatic function in *Lyve-1^wt/Cre^;Vegfr2^flox/flox^* mice. Evans blue dye (EBD) was injected into the hind paws of *Vegfr2^flox/flox^* and *Lyve-1^wt/Cre^;Vegfr2^flox/flox^* mice and was rapidly transported to the popliteal and iliac lymph nodes in both strains of mice (Figure 4; n = 6 of each genotype). EBD dye did not reflux into the mesenteric lymph nodes of *Lyve-1^wt/Cre^;Vegfr2^flox/flox^* mice, indicating that lymphatic valves functioned properly in these mice. Additionally, *Lyve-1^wt/Cre^;Vegfr2^flox/flox^* mice did not exhibit lymphedema, chylous ascites or chylothorax.

### Lyve-1^wt/Cre^;Vegfr2^flox/flox^ embryos display lymphatic hypoplasia

**Figure 5:**
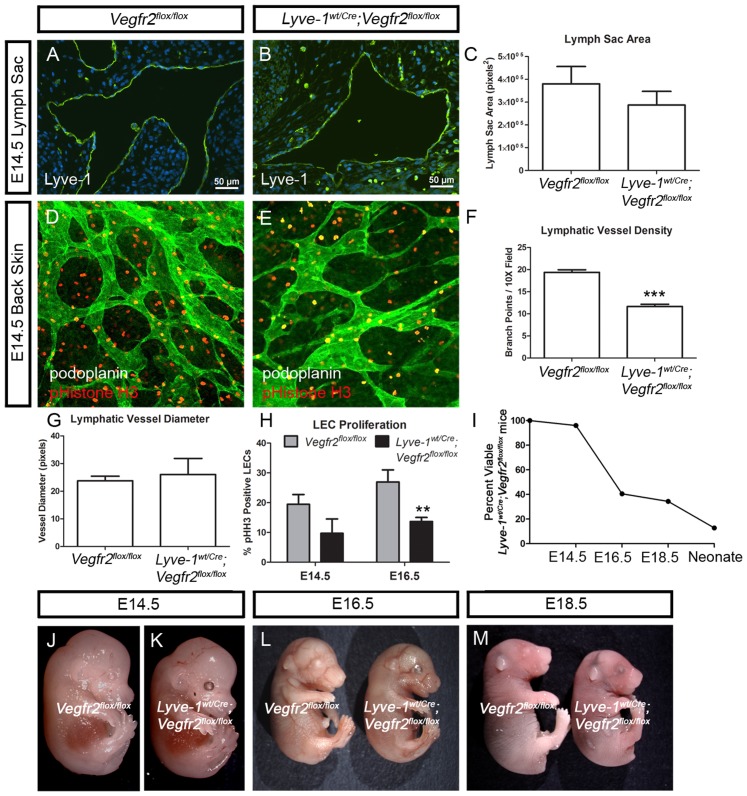
*Lyve1^wt/Cre^;Vegfr2^flox/flox^* embryos exhibit lymphatic defects. (A,B) Immunofluorescence staining for Lyve-1 showing jugular lymph sacs in E14.5 *Vegfr2^flox/flox^* and *Lyve-1^wt/Cre^;Vegfr2^flox/flox^* embryos. (C) Graph showing that lymph sac area is not different between *Vegfr2^flox/flox^* (380,110±76,279 pixels^2^; n = 8) and *Lyve-1^wt/Cre^;Vegfr2^flox/flox^* embryos (287,350±59,931 pixels^2^; n = 5). (D,E) Images of back skin from E14.5 *Vegfr2^flox/flox^* and *Lyve-1^wt/Cre^;Vegfr2^flox/flox^* embryos stained with antibodies against podoplanin (green) and phospho-histone H3 (red). (F) At E14.5, there are significantly fewer lymphatic branch points in *Lyve-^wt/Cre^;Vegfr2^flox/flox^* embryos (11.63±0.5239; n = 3) than in *Vegfr2^flox/flox^* embryos (19.37±0.5783; n = 3). (G) At E14.5, lymphatic vessel diameter is not significantly different between *Lyve-1^wt/Cre^;Vegfr2^flox/flox^* (26.00±5.859 pixels; n = 3) and *Vegfr2^flox/flox^* embryos (23.75±1.652 pixels; n = 4). (H) Graph showing that there are fewer proliferating lymphatic endothelial cells in *Lyve-1^wt/Cre^;Vegfr2^flox/flox^* embryos than in *Vegfr2^flox/flox^* embryos at E14.5 and E16.5. At E14.5, 19.45% of LECs in *Vegfr2^flox/flox^* embryos (n = 4) were phospho-Histone H3-positive whereas 9.73% of LECs in *Lyve-1^wt/Cre^;Vegfr2^flox/flox^* embryos (n = 4) were phospho-histone H3-positive. At E16.5, 26.94% of LECs in *Vegfr2^flox/flox^* embryos (n = 5) were phospho-Histone H3-positive whereas 13.69% of LECs in *Lyve-1^wt/Cre^;Vegfr2^flox/flox^* embryos (n = 7) were phospho-histone H3-positive. (I) Graph showing the percent of viable *Lyve-1^wt/Cre^;Vegfr2^flox/flox^* mice at different developmental stages. (J-M) Images of non-edematous E14.5 (J,K), E16.5 (L) and E18.5 (M) *Vegfr2^flox/flox^* and *Lyve-1^wt/Cre^;Vegfr2^flox/flox^* embryos. ** indicates P || 0.01; *** indicates P || 0.001.

Because adult *Lyve-1^wt/Cre^;Vegfr2^flox/flox^* mice display lymphatic hypoplasia, we characterized lymphatic development in *Lyve-1^wt/Cre^;Vegfr2^flox/flox^* embryos. First, we measured the size of the jugular lymph sacs in *Vegfr2^flox/flox^* and *Lyve-1^wt/Cre^;Vegfr2^flox/flox^* embryos. At E14.5, the cross-sectional area of the jugular lymph sacs was not significantly different between *Vegfr2^flox/flox^* (380,110±76,279 pixels^2^; n = 8) and *Lyve-1^wt/Cre^;Vegfr2^flox/flox^* embryos (287,350±59,931 pixels^2^; n = 5**;** Figure 5A–C). This suggests that Vegfr2 is not required for the development of lymph sacs. Next, we performed whole-mount immunofluorescence staining of skin to assess the patterning of the lymphatic vasculature in E14.5 *Vegfr2^flox/flox^* and *Lyve-1^wt/Cre^;Vegfr2^flox/flox^* embryos. This analysis revealed that the density of lymphatic vessels was significantly lower in *Lyve-1^wt/Cre^;Vegfr2^flox/flox^* embryos (11.63±0.5239; n = 3) than in *Vegfr2^flox/flox^* embryos (19.37±0.5783; n = 3; Figure 5D-F). However, the diameter of lymphatic vessels was not significantly different between *Lyve-1^wt/Cre^;Vegfr2^flox/flox^* (26.00±5.859 pixels; n = 3) and *Vegfr2^flox/flox^* embryos (23.75±1.652 pixels; n = 4; Figure 5G). To determine whether the reduction in lymphatic vessel density was due to decreased proliferation of LECs, we stained tissues for podoplanin and phospho-histone H3. We observed that the number of proliferating LECs was lower in *Lyve-1^wt/Cre^;Vegfr2^flox/flox^* embryos than in *Vegfr2^flox/flox^* embryos at E14.5 and E16.5 (Figure 5H). However, this reduction was only statistically significant at E16.5. These findings indicate that Vegfr2 is required for the proper growth and expansion of lymphatic vessels during embryonic development.

### Lyve-1^wt/Cre^;Vegfr2^flox/flox^ embryos display reduced viability and have fewer blood vessels in the yolk sac, liver and lung than Vegfr2^flox/flox^ embryos

**Figure 6:**
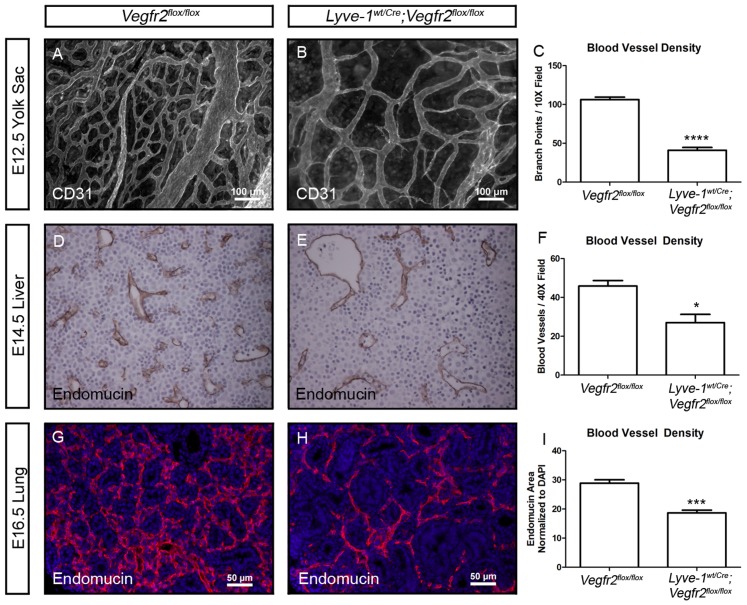
The density of blood vessels is reduced in the yolk sac, liver and lung in *Lyve-1^wt/Cre^;Vegfr2^flox/flox^* embryos. (A,B) Whole-mount immunofluorescence staining of yolk sacs from E12.5 *Vegfr2^flox/flox^* and *Lyve-1^wt/Cre^;Vegfr2^flox/flox^* embryos for CD31. (C) There are significantly fewer blood vessel branch points in yolk sacs from *Lyve-1^wt/Cre^;Vegfr2^flox/flox^* embryos (41.06±3.662, n = 4 embryos) than *Vegfr2^flox/flox^* embryos (106.3±3.157, n = 3 embryos). (D-F) Immunohistochemical staining of E14.5 livers for endomucin showing significantly fewer blood vessels in *Lyve-1^wt/Cre^;Vegfr2^flox/flox^* embryos (27.00±4.263, n = 4 embryos) than in *Vegfr2^flox/flox^* embryos (45.88±2.850, n = 4 embryos). (G-I) Immunofluorescence staining of E16.5 lungs for endomucin (red) and DAPI (blue) showing that the density of blood vessels is lower in *Lyve-1^wt/Cre^;Vegfr2^flox/flox^* (18.67±0.962; n = 5) embryos than in *Vegfr2^flox/flox^* embryos (28.89±1.145; n = 5). * indicates P || 0.05; *** indicates P || 0.001; **** indicates P || 0.0001.

Crosses between *Vegfr2^flox/flox^* and *Lyve-1^wt/Cre^;Vegfr2^wt/flox^* mice revealed that most *Lyve-1^wt/Cre^;Vegfr2^flox/flox^* mice die after E14.5 (Table 1 and Figure 5I). Importantly, *Lyve-1^wt/Cre^;Vegfr2^flox/flox^* embryos were not edematous at E14.5 or at later time points (Figure 5J-M). However, *Lyve-1^wt/Cre^;Vegfr2^flox/flox^* embryos tended to be smaller than their wildtype littermates. Therefore, we examined *Lyve-1^wt/Cre^;Vegfr2^flox/flox^* embryos for a potential cardiovascular defect. Lyve-1 has recently been reported to be expressed by BECs in the yolk sac, liver and lung [36]. Indeed, crosses with *mT/mG* reporter mice demonstrated that *Lyve-1^wt/Cre^* mice display *Cre* recombinase activity in BECs in these tissues (Figure S1). To determine whether the vasculature was altered in these tissues, we performed immunohistochemistry with markers of BECs. Whole-mount immunofluorescence staining for CD31 revealed that there were significantly fewer blood vessel branch points in E12.5 *Lyve-1^wt/Cre^;Vegfr2^flox/flox^* yolk sacs (41.06±3.662, n = 4) than in E12.5 *Vegfr2^flox/flox^* yolk sacs (106.3±3.157, n = 3) (Figure 6A–C). Immunohistochemistry for endomucin showed that there were significantly fewer blood vessels in E14.5 *Lyve-1^wt/Cre^;Vegfr2^flox/flox^* livers (27.00±4.263, n = 4) than in E14.5 *Vegfr2^flox/flox^* livers (45.88±2.850, n = 4) (Figure 6D-F). Additionally, immunofluorescence staining revealed that the density of blood vessels was significantly lower in E16.5 *Lyve-1^wt/Cre^;Vegfr2^flox/flox^* lungs (18.67±0.962; n = 5) than in E16.5 *Vegfr2^flox/flox^* lungs (28.89±1.145; n = 5; Figure 6G-I). These observations reveal that the loss of Vegfr2 in BECs in the yolk sac, liver and lung severely impairs vascular development in these tissues.

**Table 1:** Observed number of mice following crosses between *Vegfr2^flox/flox^* and *Lyve-1^wt/Cre^;Vegfr2^flox/flox^* mice.

Genotype	E12.5	E14.5	E16.5	E18.5	Neonates
*Lyve-1^wt/wt^;Vegfr2^wt/flox^*	6	16	19	21	72
*Lyve-1^wt/wt^;Vegfr2^flox/flox^*	4	19	20	19	67
*Lyve-1^wt/Cre^;Vegfr2^wt/flox^*	8	14	23	13	72
*Lyve-1^wt/Cre^;Vegfr2^flox/flox^*	6	16	7	5	7
Total number of mice	24	65	69	58	218

### Lyve-1^wt/Cre^;Vegfr2^flox/flox^ embryos do not display a heart defect

Endocardium has been reported to express Lyve-1 throughout embryogenesis [36]. Therefore, we examined *Lyve-1^wt/Cre^;Vegfr2^flox/flox^* embryos for a heart defect. An embryo with poor cardiac function will usually display pericardial edema and a blood-engorged liver [37]. Importantly, *Lyve-1^wt/Cre^;Vegfr2^flox/flox^* embryos did not exhibit pericardial edema (n = 9) or blood-engorged livers (n = 4; Figure S2). Additionally, *Lyve-1^wt/Cre^;Vegfr2^flox/flox^* embryos did not display obvious structural defects of the heart at E14.5 (n = 4) or E16.5 (n = 5; Figure S2). Staining for endomucin did not reveal differences in the integrity of the endocardium between *Vegfr2^flox/flox^* and *Lyve-1^wt/Cre^;Vegfr2^flox/flox^* embryos (Figure S2). This prompted us to characterize the expression of Lyve-1 in the heart. We stained hearts from E16.5 wildtype embryos for Lyve-1 and found that it was expressed in a faint scattered pattern by the endocardium (Figure S3). Our observation is in agreement with Gordon et al., (2008), who reported a “salt and pepper” pattern of Lyve-1 expression by the endocardium [36]. This reduced expression pattern for Lyve-1 may result in low levels of Cre recombinase and explain why the endocardium is not compromised in *Lyve-1^wt/Cre^;Vegfr2^flox/flox^* embryos. Together, these findings suggest that *Lyve-1^wt/Cre^;Vegfr2^flox/flox^* embryos do not suffer from a heart defect.

### Vegfr2 is not expressed by macrophages and inactivation of Vegfr2 in the myeloid lineage does not affect lymphatic development

**Figure 7:**
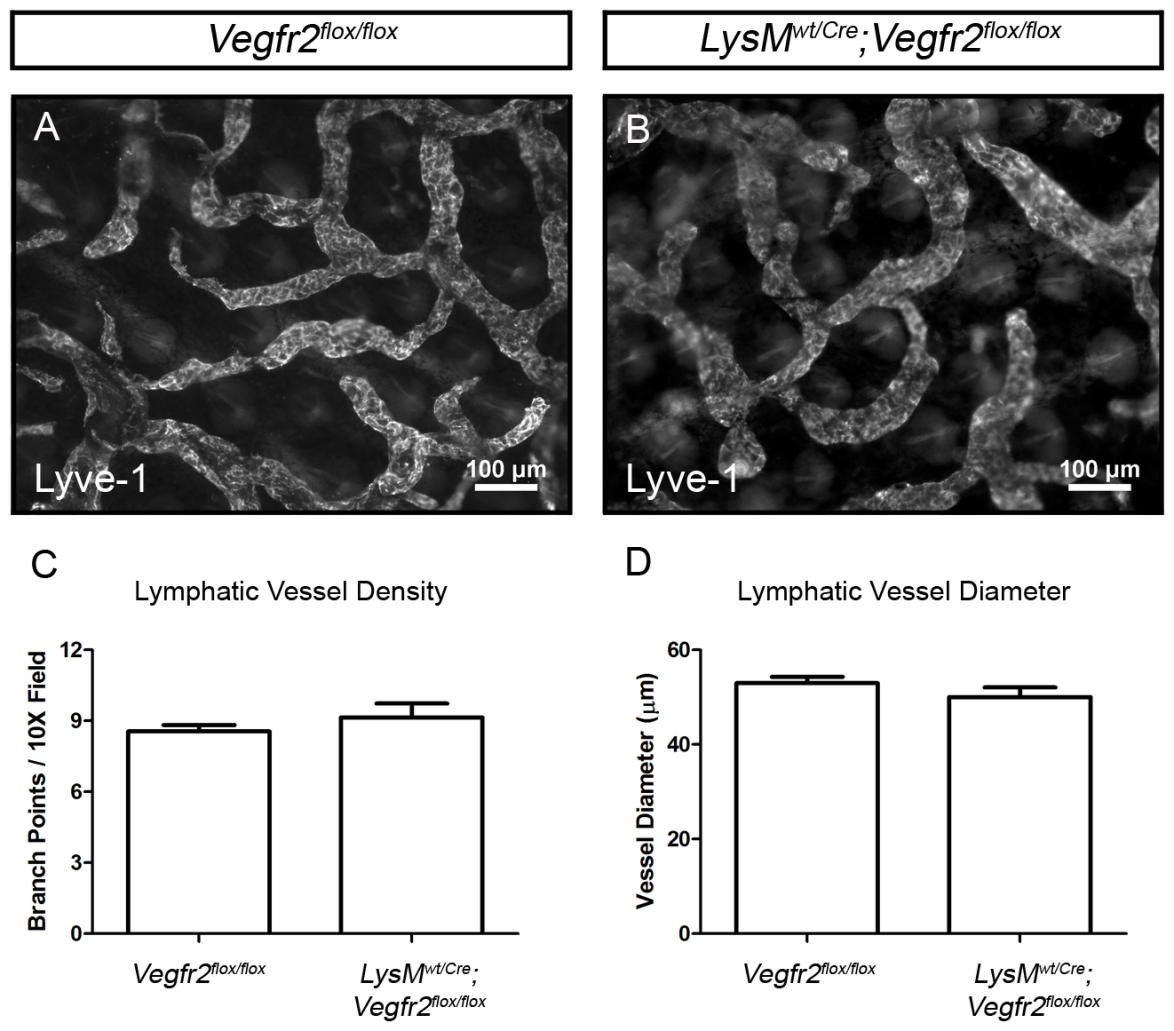
Deleting *Vegfr2* in the myeloid lineage does not affect the development of the lymphatic vascular system. (A,B) Whole-mount immunofluorescence staining of ear skin from *Vegfr2^flox/flox^* and *LysM^wt/Cre^;Vegfr2^flox/flox^* mice for Lyve-1. (C) Quantitative analysis showing that the number of lymphatic branch points in the ear skin of *Vegfr2^flox/flox^* (8.550±0.278, n = 5 mice) and *LysM^wt/Cre^;Vegfr2^flox/flox^* mice (9.150±0.5895, n = 5 mice) are not significantly different from one another. (D) Furthermore, lymphatic vessel diameter is not significantly different between *Vegfr2^flox/flox^* (52.98 µm±1.328, n = 5 mice) and *LysM^wt/Cre^;Vegfr2^flox/flox^* mice (50.05 µm±2.031, n = 4 mice).

Crosses with *mT/mG* reporter mice revealed that *Lyve-1^Cre^* mice exhibit *Cre* recombinase activity in macrophages as well as lymphatic vessels (Figure 1A, 1B). Therefore, several experiments were performed to rule out the possibility that the lymphatic phenotype of *Lyve-1^wt/Cre^;Vegfr2^flox/flox^* mice was due to the inactivation of *Vegfr2* in macrophages. First, we explored the expression of Vegfr2 by macrophages. Whole-mount immunofluorescence staining showed that Vegfr2 was not expressed by macrophages in the ear skin of *Vegfr2^wt/GFP^* mice (Figure S4). Next, the *LysM^Cre^* strain was used to conditionally delete target sequences in the myeloid lineage. *LysM^Cre^* mice were bred with *mT/mG* reporter mice to characterize the expression pattern of *Cre* recombinase and to trace the fate of cells of the myeloid lineage. All *LysM^wt/Cre^;mT/mG* mice displayed strong GFP expression by macrophages in the ear skin (Figure S5). GFP did not co-localize with Vegfr3 in the ear skin of *LysM^wt/Cre^;mT/mG* mice (Figure S5; n = 5 mice). This finding indicates that cells genetically marked by *LysM^Cre^* do not differentiate into LECs during normal murine development and is in agreement with another report using the *LysM^Cre^* line [38]. *LysM^Cre^* mice were then crossed with *Vegfr2^flox^* mice to determine whether deleting *Vegfr2* in myeloid cells affects the development of the lymphatic vasculature. Whole-mount immunofluorescence staining of ear skin for Lyve-1 revealed that the density of lymphatic vessels was not significantly different between *Vegfr2^flox/flox^* (8.550±0.278, n = 5 mice) and *LysM^wt/Cre^;Vegfr2^flox/flox^* (9.150±0.5895, n = 5 mice) littermates (Figure 7A-D). Furthermore, the diameter of lymphatic vessels was not significantly different between *Vegfr2^flox/flox^* (52.98 µm±1.328, n = 5 mice) and *LysM^wt/Cre^;Vegfr2^flox/flox^* (50.05 µm±2.031, n = 4 mice) littermates (Figure 7A-D). EBD was also effectively transported from injected hind paws to popliteal and iliac lymph nodes in all *Vegfr2^flox/flox^* and *LysM^wt/Cre^;Vegfr2^flox/flox^* mice (data not shown). Together, these data reveal that *Vegfr2* is not required in the myeloid lineage for the proper development of the lymphatic system. This demonstrates that the lymphatic phenotype of *Lyve-1^wt/Cre^;Vegfr2^flox/flox^* mice is due to the ablation of *Vegfr2* in LECs, not macrophages.

## Discussion

VEGFR2 is widely recognized as an essential gene driving the formation of the blood vasculature during embryogenesis. The present study demonstrates that VEGFR2 also directly promotes the development of the lymphatic vasculature. We show that the density, but not diameter, of lymphatic vessels is dramatically reduced in *Lyve-1^wt/Cre^;Vegfr2^flox/flox^* mice. Additionally, we demonstrate that lymphatic vessels in *Lyve-1^wt/Cre^;Vegfr2^flox/flox^* mice properly mature into collecting vessels. These findings indicate that VEGFR2 is required for the expansion, but not the specification or maturation, of the lymphatic vasculature.

The lymphatic vasculature of mammals can grow by undergoing sprouting lymphangiogenesis, in which new lymphatic vessels emerge from pre-existing vessels, or circumferential lymphangiogenesis, which is characterized by an increase in the diameter of lymphatic vessels [19]. Sprouting lymphangiogenesis increases the number of lymphatic branch points and complexity of the lymphatic network. Therefore, if VEGFR2 stimulates sprouting lymphangiogenesis, there should be fewer lymphatic branch points in *Lyve-1^wt/Cre^;Vegfr2^flox/flox^* mice than in *Vegfr2^flox/flox^* mice. On the other hand, if VEGFR2 promotes circumferential lymphangiogenesis, lymphatic vessels should have a smaller diameter in *Lyve-1^wt/Cre^;Vegfr2^flox/flox^* mice than in *Vegfr2^flox/flox^* mice. Importantly, we found that the number of lymphatic branch points was greatly reduced in *Lyve-1^wt/Cre^;Vegfr2^flox/flox^* mice and that the width of lymphatic vessels was not significantly different between wildtype and mutant mice. In contrast to a previous report [19], our results suggest that VEGFR2 signaling promotes sprouting lymphangiogenesis rather than circumferential lymphangiogenesis. The earlier model proposing that VEGFR2 stimulates circumferential rather than sprouting lymphangiogenesis was based on experiments showing that adenoviral and transgenic overexpression of VEGF-E (VEGFR2-specific ligand) preferentially induces the enlargement of lymphatic vessels instead of the formation of new lymphatic vessels [19]. The discrepancy between our findings and this previous report could be due to the fact that overexpression of VEGF-E increases blood vessel permeability [19], an effect which may contribute to the enlargement of lymphatic vessels. Alternatively, the effect of VEGF-E on lymphatics may be unique to this factor, as adenoviral expression of VEGF-A^164^ induces lymphatic vessel sprouting *in vivo*, albeit to a lesser extent than VEGF-C [19]. Furthermore, VEGF-A has also been shown to induce sprouting lymphangiogenesis in mouse corneas and *in vitro* by LEC spheroids [21], [39]. Together, these observations suggest that the function of VEGFR2 in lymphatic vessels is similar to its function in blood vessels, where it also stimulates the budding of new vessels from pre-existing vessels [40].

Although it is well established that lymphatic vessels can grow by sprouting, the underlying mechanism of sprouting lymphangiogenesis is not well defined. In contrast, the process of sprouting hemangiogenesis is well characterized. Growing blood vessels are led by specialized tip cells which migrate and extend numerous filopodia to probe the microenvironment for directional cues [40]. These cells are followed by stalk cells that proliferate in response to growth factors and thereby promote vessel extension [40]. VEGF-A/VEGFR2 signaling plays a critical role in regulating tip cell activity (migration/filopodia extension) and stalk cell proliferation [41]. VEGFR2 may also control sprouting lymphangiogenesis in a similar fashion. *In vivo*, VEGFR2 is expressed by growing lymphatic vessels and their filopodia [42]. Whether the LECs extending filopodia are true tip cells has yet to be determined. Nevertheless, VEGFR2 at the end of a growing lymphatic vessel could be involved in sensing directional signals and migration. Additionally, VEGFR2 could promote vessel extension by stimulating the proliferation of LECs. We found that LEC proliferation is reduced in *Lyve-1^wt/Cre^;Vegfr2^flox/flox^* embryos. Furthermore, we and others have previously shown that VEGF-A/VEGFR2 signaling promotes LEC migration and proliferation *in vitro* [28]. Therefore, the loss of VEGFR2 in LECs in *Lyve-1^wt/Cre^;Vegfr2^flox/flox^* mice may impair lymphangiogenesis by affecting the migration and proliferation of LECs.

During development specific lymphatic vessels acquire a collecting vessel phenotype, a process involving the recruitment of mural cells and formation of intraluminal valves. Recent studies of genetically modified mice have identified several genes that participate in collecting vessel maturation, such as Ang2, Ephrinb2, and NFATC1 [30]–[32], [43], [44]. Importantly, VEGFR2 signaling induces the expression of Ang2, nuclear translocation of NFATC1, and is regulated by Ephrinb2-mediated internalization and trafficking [24], [32], [45]. These observations, as well as the high expression of VEGFR2 by collecting vessels and valves ([19] and data not shown), led us to test the hypothesis that VEGFR2 participates in the maturation of lymphatics into collecting vessels. To our surprise we found that *Lyve-1^wt/Cre^;Vegfr2^flox/flox^* mice develop normal collecting lymphatic vessels. Mural cells were properly associated with lymphatic vessels and intraluminal valves were present in *Lyve-1^wt/Cre^;Vegfr2^flox/flox^* mice. The lack of an abnormal collecting vessel phenotype may be due to compensation by VEGFR3, which is also expressed by collecting vessels and valves [19]. Future work with VEGFR3 and VEGFR2 mutant mice will help elucidate the role these receptors serve in the remodeling of the lymphatic system.

During the course of our study we discovered that most *Lyve-1^wt/Cre^;Vegfr2^flox/flox^* mice die during embryonic development. Previously described mutant embryos that display fatal lymphatic defects die from edema [15], [46]. However, *Lyve-1^wt/Cre^;Vegfr2^flox/flox^* embryos were not edematous at any of the time points analyzed. This suggests that *Lyve-1^wt/Cre^;Vegfr2^flox/flox^* embryos do not die from a lymphatic defect. Therefore, the *Lyve-1^Cre^* allele must induce the loss of Vegfr2 in a different cell type that is required for survival. In agreement with a previous study that documented Lyve-1 expression in blood vessels of the yolk sac, liver and lung [36], we found that *Lyve-1^Cre^* mice expressed *Cre* recombinase in blood vessels of the yolk sac, liver and lung. We also found that the density of blood vessels was dramatically reduced in these tissues in *Lyve-1^wt/Cre^;Vegfr2^flox/flox^* embryos. Proper development of the yolk sac vasculature is required for mice to survive to birth [37]. Therefore, *Lyve-1^wt/Cre^;Vegfr2^flox/flox^* embryos may die of a yolk sac vascular defect. Additionally, vascular defects in the liver and lung may also contribute the lethal phenotype of *Lyve-1^wt/Cre^;Vegfr2^flox/flox^* mice. Select *Lyve-1^wt/Cre^;Vegfr2^flox/flox^* mice may survive because they display low *Cre* recombinase activity in BECs in the yolk sac, liver and lung. Alternatively, differences in genetic background may contribute to the survival of a subset of *Lyve-1^wt/Cre^;Vegfr2^flox/flox^* mice.

In conclusion, we show that VEGFR2 directly promotes the expansion of the lymphatic vessel network. This newly identified function of VEGFR2 further defines the molecular pathways controlling the development of the lymphatic vasculature and sheds light on the mechanisms by which therapeutic agents targeting VEGFR2 inhibit lymphangiogenesis.

## Materials and Methods

### Ethics Statement

Experiments performed with mice were carried out in accordance with an animal protocol approved by the IACUC of the University of Texas Southwestern Medical Center (APN 0974-07-05-1).

### Mice and genotyping

*Vegfr2^flox^* (Balb/c genetic background) [47], *mT/mG* (mixed genetic background) [34], *Lyve-1^Cre^* (129 and C57Bl/6 mixed genetic background) [33], *LysM^Cre^* (C57Bl/6 genetic background) [48] and *Vegfr2^GFP^* (CD1 genetic background) [49] mice have been described previously. *Vegfr2^flox^* mice were genotyped with the following primers: 5′-TGG-AGA-GCA-AGG-CGC-TGC-TAG-C-3′ and 5′-CTT-TCC-ACT-CCT-GCC-TAC-CTA-G-3′ to yield a 322 bp wildtype band and a 439 bp mutant band. *mT/mG* mice were genotyped with the following primers: 5′-CTC-TGC-TGC-CTC-CTG-GCT-TCT-3′, CGA-GGC-GGA-TCA-CAA-GCA-ATA-3′, and 5′-TCA-ATG-GGC-GGG-GGT-CGT-T-3′ to yield a 330 bp wildtype band and a 250 bp mutant band. *Lyve-1^Cre^* mice were genotyped with the following primers: 5′-TGC-CAC-CTG-AAG-TCT-CTC-CT- 3′, 5′-TGA-GCC-ACA-GAA-GGG-TTA-GG-3′, and 5′-GAG-GAT-GGG-GAC-TGA-AAC-TG-3′ to yield a 425 bp wildtype band and a 210 bp mutant band. *LysM^Cre^* mice were genotyped with the following primers: 5′-CCC-AGA-AAT-GCC-AGA-TTA-CG-3′, 5′-TTA-CAG-TCG-GCC-AGG-CTG-AC-3′, and 5′-CTT-GGG-CTG-CCCA-GAA-TTT-CTC-3′ to yield a 700 bp wildtype band and a 350 bp mutant band.

### Antibodies

The following primary antibodies were used for immunohistochemistry or immunofluorescence staining of mouse tissues: rabbit anti-Lyve-1 (abcam, cat no. ab14917), goat anti-Lyve-1 (R&D Systems, cat no. AF2125), rat anti-endomucin (Santa Cruz, cat no. sc-65495), rat anti-CD31 (BD Biosciences, cat no. 550300), Cy3-conjugated mouse anti-smooth muscle actin (Sigma, cat no. C6198), hamster anti-podoplanin (abcam, cat no. ab11936), rat anti-Vegfr3 (eBioscience, 14-5988-81), rabbit anti-phospho-histone H3 (Millipore, cat no. 06-570) and rabbit anti-Vegfr2 (T014, purified in our laboratory [50]). All secondary antibodies were from Jackson ImmunoResearch.

### Whole-mount immunofluorescence staining

Tissues were fixed overnight at 4°C in 1% PFA, washed with PBS (6 X 15 minutes), permeabilized for 1 hour with PBS + 0.3% Triton-X 100, then blocked with overnight at 4°C in PBS + 0.3% Triton-X 100 + 20% Aquablock (East Coast Biologics, cat no. PP82-P0691). Tissues were then incubated overnight at 4°C with primary antibodies diluted in PBS + 0.3% Triton-X 100. Following this step, tissues were washed with PBS + 0.3% Triton-X 100 (3 X 40 minutes), incubated overnight at 4°C with the appropriate secondary antibodies, then washed again with PBS + 0.3% Triton-X 100 (3 X 40 minutes). Coverslips were mounted with ProLong Gold plus DAPI (Invitrogen, P36931). Slides were analyzed using a Nikon Eclipse E600 microscope and images captured using NIS-Elements imaging software.

### Quantitative analysis of lymphatic branch points and diameter

For adult mice, the number of branches was counted in 4 images from each mouse at 10X magnification. For embryos, the number of branches was counted in 1-6 images from each mouse at 10X magnification. The same images were used to assess lymphatic vessel diameter. A grid with lines spaced 75μm from one another was placed over each 10X image. Vessel diameter was measured with the NIS-Elements imaging software at locations where two perpendicular grid lines intersected a lymphatic vessel.

### Immunofluorescence and immunohistochemical staining of tissue sections

Embryos were fixed overnight at 4°C in 4% PFA, washed with 50% EtOH, processed, and then sectioned at 5 µm for staining. Slides were deparaffinized with xylene and rehydrated through a descending EtOH series. Antigen retrieval was performed with 0.01 M citric acid (pH = 6.0) in a pressure cooker. Slides were then washed with PBS and blocked for 1 hour with TBST + 20% Aquablock. Primary antibodies diluted in TBST + 5% BSA were then added and allowed to incubate overnight at 4°C. Slides were washed with TBST then secondary antibodies diluted in TBST + 5% BSA were added and allowed to incubate for 1 hour at room temperature. Slides were then washed again with TBST and coverslips were mounted with ProLong Gold plus DAPI. Immunohistochemistry was performed with using a similar protocol except endogenous peroxidase activity was blocked by incubating slides with hydrogen peroxide diluted in MeOH and signal was detected via the DAB chromogen system (Dako, cat no. K3468).

### Evans blue dye lymphangiography

The popliteal and iliac regions were examined for lymph transport following the injection of Evans blue dye (1% w/v) into the hind paws of mice anesthetized with isoflurane and kept warm with a heating pad.

### Assessment of Vegfr2 expression by lymphatic vessels

Frozen sections of adult ear skin were stained with antibodies against Lyve-1 and Vegfr2. Four images at 20X magnification were taken of each section of ear skin. The number of Vegfr2-positive and Vegfr2-negative LECs in each image was manually counted. The number of Vegfr2-positive LECs was divided by the total number of LECs and then multiplied by 100 to determine the percent of Vegfr2-positive LECs.

### Assessment of LEC proliferation

Tissue sections of E14.5 and E16.5 embryos were stained with antibodies against podoplanin and phospho-histone H3. Six images at 40X magnification were taken of each tissue section. The number of phospho-histone H3-positive and phospho-histone H3-negative LECs in each image was manually counted. The number of phospho-histone H3-positive LECs was divided by the total number of LECs and then multiplied by 100 to determine the percent of LECs proliferating.

### Statistical analysis

Data were analyzed using GraphPad Prism statistical analysis software (Version 5.0). All results are expressed as mean ± SEM. Unpaired student’s T-tests were performed to test means for significance. Data were considered significant at *P*<0.05.

## Supporting Information

Figure S1 ***Lyve-1^Cre^*** **is expressed by blood endothelial cells in the yolk sac, liver and lung.** Representative images of *Lyve-1^wt/Cre^;mT/mG* tissues showing GFP expression by blood endothelial cells in the yolk sac, liver and lungs.
(TIF)

Figure S2 ***Lyve-1^wt/Cre^;Vegfr2^flox/flox^*** **embryos do not display a cardiac defect.** (A,B) H & E stained sections of E14.5 *Vegfr2^flox/flox^* and *Lyve-1^wt/Cre^;Vegfr2^flox/flox^* embryos. (C,D) Endomucin immunolabeled sections of E16.5 *Vegfr2^flox/flox^* and *Lyve-1^wt/Cre^;Vegfr2^flox/flox^* embryos. Hearts appear normal and pericardial edema is not present in E14.5 or E16.5 *Lyve-1^wt/Cre^;Vegfr2^flox/flox^* embryos. (E,F) H & E stained sections of livers from E14.5 *Vegfr2^flox/flox^* and *Lyve-1^wt/Cre^;Vegfr2^flox/flox^* embryos.
(TIF)

Figure S3 **Lyve-1 is not strongly expressed by endocardium in wildtype embryos.** Endomucin is strongly expressed by blood endothelial cells in the lung and by endocardium (arrow). In contrast, Lyve-1 is strongly expressed by blood endothelial cells in the lung but not by endocardium (arrow). Lyve-1 was present in a faint “salt and pepper” pattern in the heart.
(TIF)

Figure S4 **Macrophages do not express Vegfr2.** (A-C) Whole-mount immunofluorescence staining showing a GFP (Vegfr2)-negative-Lyve-1-positive macrophage in the ear skin of a *Vegfr2^wt/GFP^* mouse.
(TIF)

Figure S5 ***LysM^Cre^*** **is not expressed by lymphatic endothelial cells.** (A,B) GFP is not expressed in ear skin from *mT/mG* mice. (C) GFP expression by macrophages is shown in a whole-mount preparation of ear skin from an adult *LysM^wt/Cre^;mT/mG* mouse. (D) GFP (green) does not co-localize with VEGFR3 (red) in the ear skin of a *LysM^wt/Cre^;mT/mG* mouse.
(TIF)
