# Supplementary figures and images for: Vascular Endothelial Growth Factor Receptor-2 Promotes the Development of the Lymphatic Vasculature
Source: PLoS One. 2013 Sep 2;8(9):e74686. doi: 10.1371/journal.pone.0074686 (PMC3759473; doi:10.1371/journal.pone.0074686)

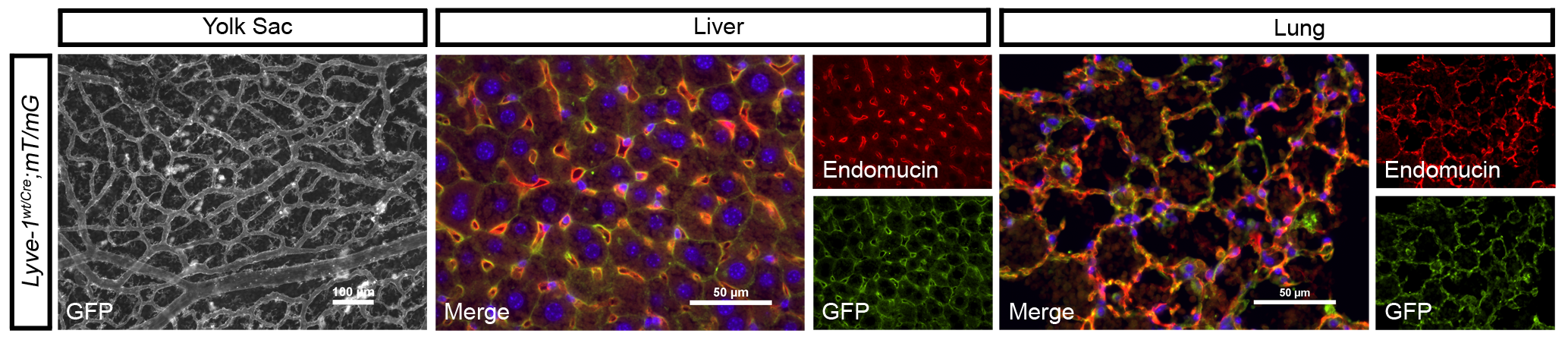

Supplement: Figure S1 — Lyve-1Cre is expressed by blood endothelial cells in the yolk sac, liver and lung. Representative images of Lyve-1wt/Cre;mT/mG tissues showing GFP expression by blood endothelial cells in the yolk sac, liver and lungs. (TIF) [file pone.0074686.s001.tif]

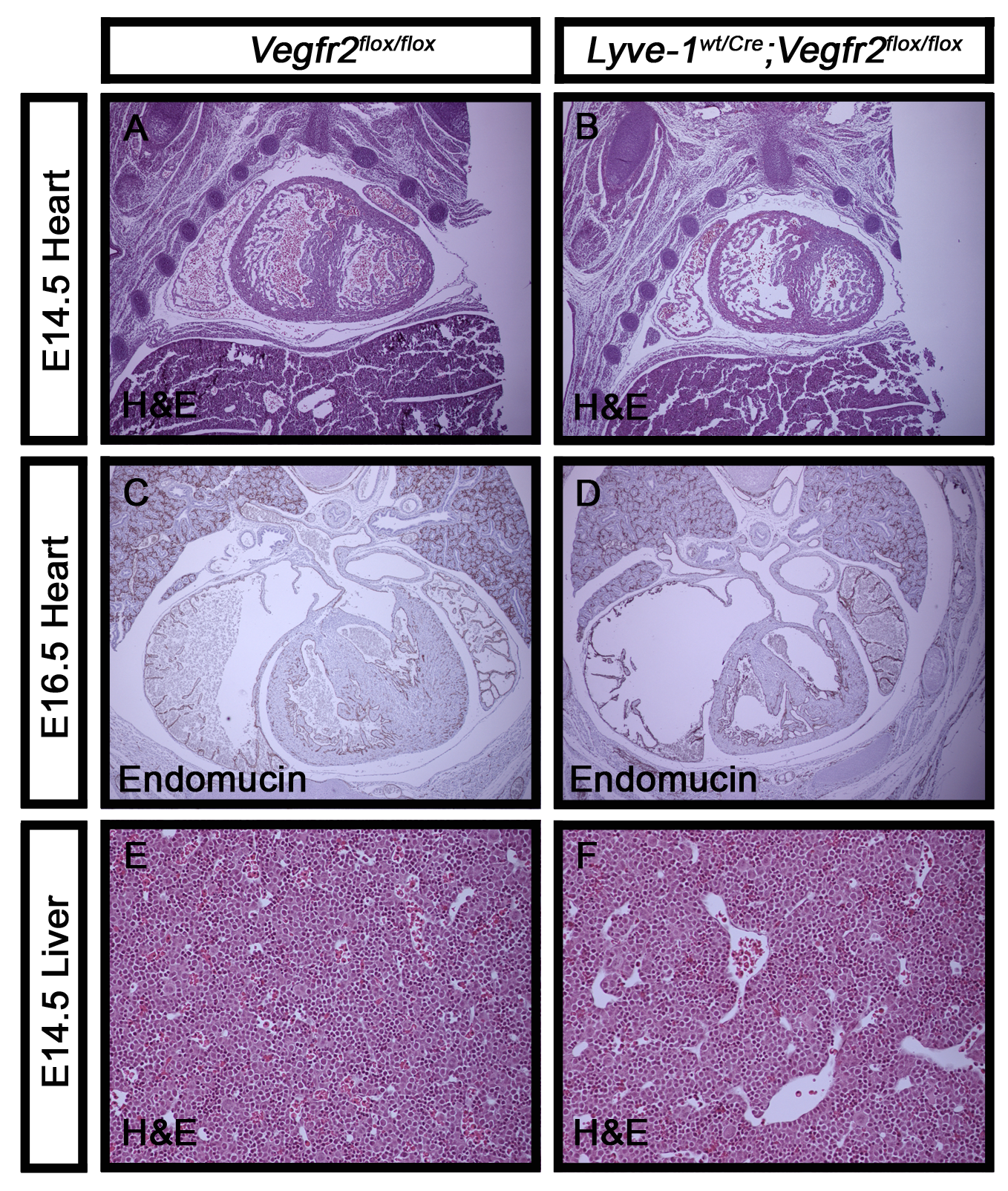

Supplement: Figure S2 — Lyve-1wt/Cre;Vegfr2flox/flox embryos do not display a cardiac defect. (A,B) H & E stained sections of E14.5 Vegfr2flox/flox and Lyve-1wt/Cre;Vegfr2flox/flox embryos. (C,D) Endomucin immunolabeled sections of E16.5 Vegfr2flox/flox and Lyve-1wt/Cre;Vegfr2flox/flox embryos. Hearts appear normal and pericardial edema is not present in E14.5 or E16.5 Lyve-1wt/Cre;Vegfr2flox/flox embryos. (E,F) H & E stained sections of livers from E14.5 Vegfr2flox/flox and Lyve-1wt/Cre;Vegfr2flox/flox embryos. (TIF) [file pone.0074686.s002.tif]

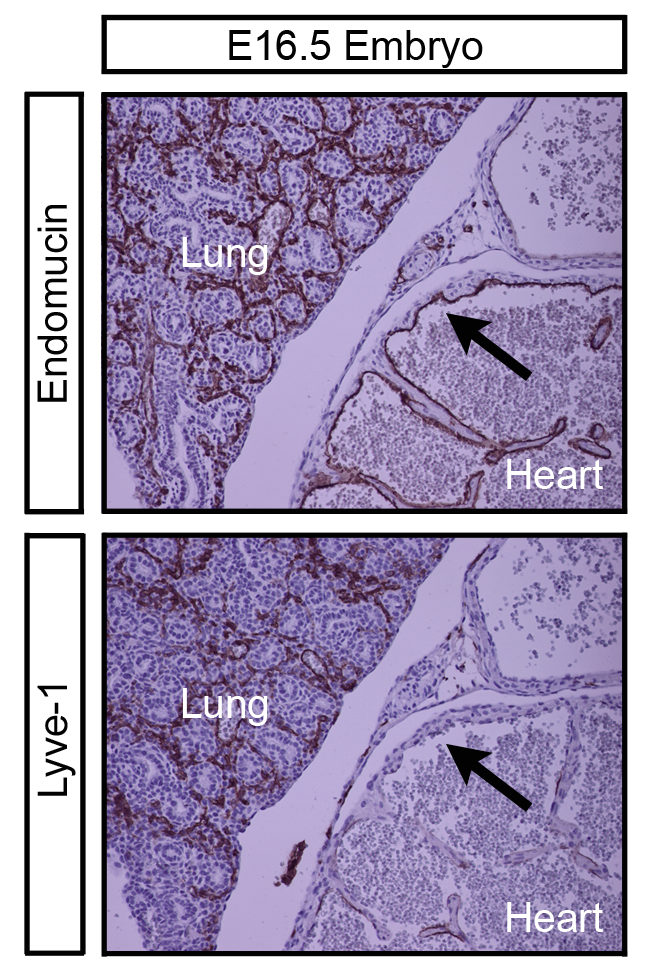

Supplement: Figure S3 — Lyve-1 is not strongly expressed by endocardium in wildtype embryos. Endomucin is strongly expressed by blood endothelial cells in the lung and by endocardium (arrow). In contrast, Lyve-1 is strongly expressed by blood endothelial cells in the lung but not by endocardium (arrow). Lyve-1 was present in a faint “salt and pepper” pattern in the heart. (TIF) [file pone.0074686.s003.tif]

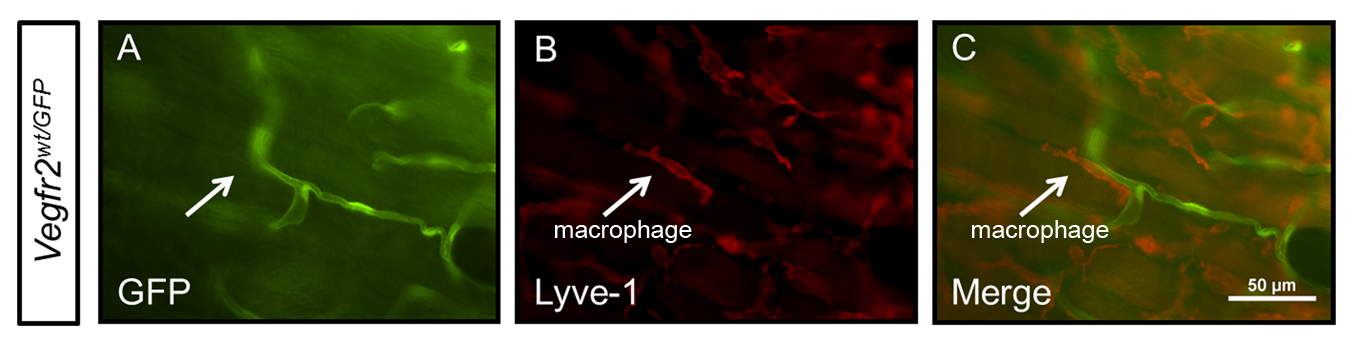

Supplement: Figure S4 — Macrophages do not express Vegfr2. (A-C) Whole-mount immunofluorescence staining showing a GFP (Vegfr2)-negative-Lyve-1-positive macrophage in the ear skin of a Vegfr2wt/GFP mouse. (TIF) [file pone.0074686.s004.tif]

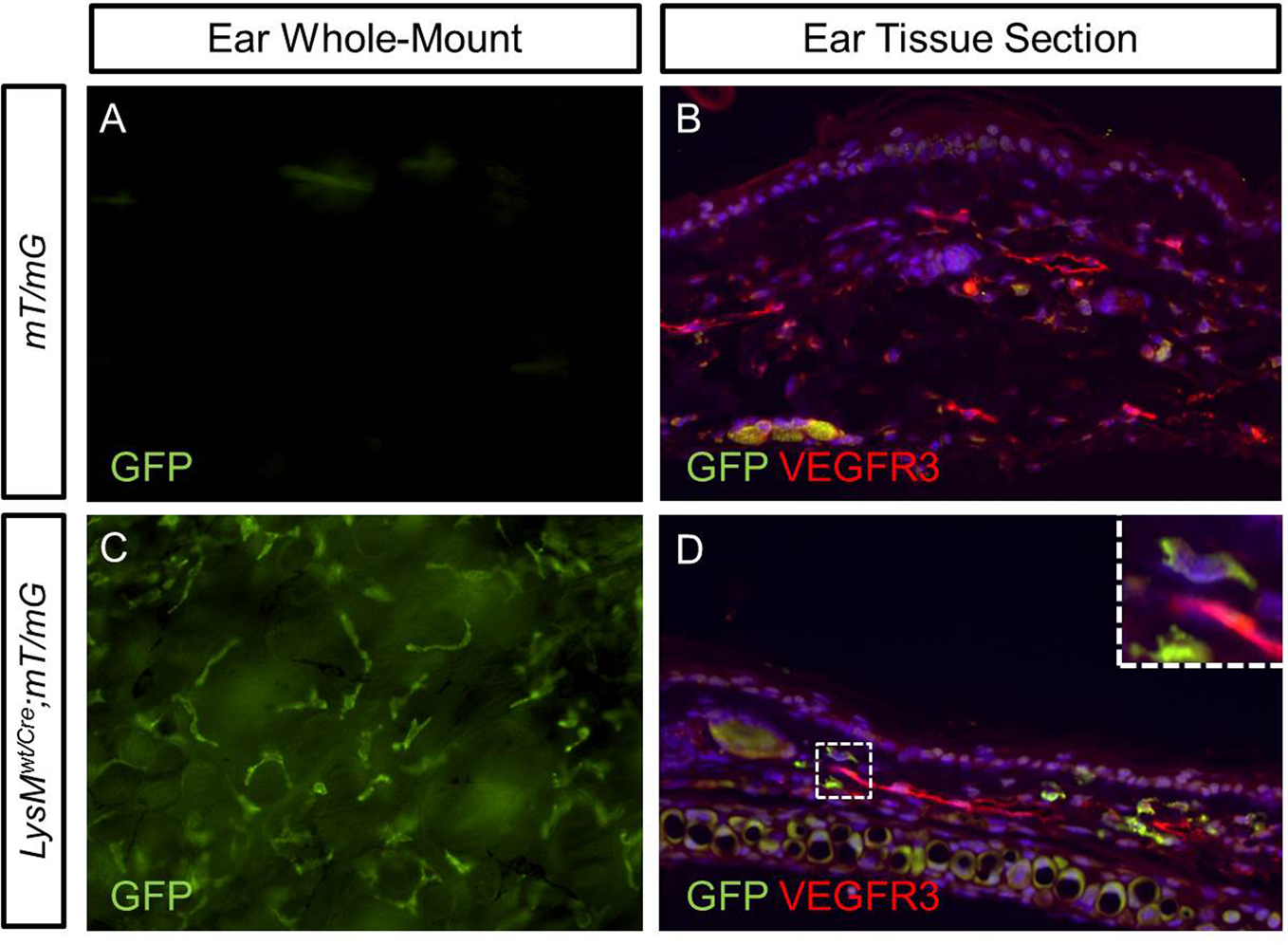

Supplement: Figure S5 — LysMCre is not expressed by lymphatic endothelial cells. (A,B) GFP is not expressed in ear skin from mT/mG mice. (C) GFP expression by macrophages is shown in a whole-mount preparation of ear skin from an adult LysMwt/Cre;mT/mG mouse. (D) GFP (green) does not co-localize with VEGFR3 (red) in the ear skin of a LysMwt/Cre;mT/mG mouse. (TIF) [file pone.0074686.s005.tif]
